# Predicting diabetic retinopathy stages using a simple nerve conduction measuring device, DPNCheck^®^: a retrospective observational study

**DOI:** 10.3389/fcdhc.2025.1590407

**Published:** 2025-07-16

**Authors:** Mayu Sakai, Takehiro Kato, Takuma Ishihara, Ken Takao, Tokuyuki Hirose, Sodai Kubota, Saki Kubota-Okamoto, Toshinori Imaizumi, Yoshihiro Takahashi, Masami Mizuno, Takuo Hirota, Yukio Horikawa, Hirokazu Sakaguchi, Shin Tsunekawa, Daisuke Yabe

**Affiliations:** ^1^ Department of Occupational Health, Gifu University Graduate School of Medicine, Gifu, Japan; ^2^ Department of Diabetes, Endocrinology and Metabolism Gifu University Graduate School of Medicine, Gifu, Japan; ^3^ Department of Rheumatology and Clinical Immunology, Gifu University Graduate School of Medicine, Gifu, Japan; ^4^ Innovative and Clinical Research Promotion Center, Gifu University Hospital, Gifu, Japan; ^5^ Department of Ophthalmology, Gifu University Graduate School of Medicine, Gifu, Japan; ^6^ Center for One Medicine Innovative Translational Research, Gifu University Institute of Advanced Studies, Gifu, Japan

**Keywords:** diabetic retinopathy, diabetic neuropathy, DPNCheck^®^, modified Baba classification, retrospective observational study

## Abstract

**Aims/introduction:**

Diabetic retinopathy (DR) often remains asymptomatic until it reaches advanced stages, when delayed treatment can lead to irreversible visual impairment. To promote timely ophthalmology visits, this study investigated the utility of a simple nerve conduction device, DPNCheck^®^, as a predictor of DR severity. Previous research has established a relationship between diabetic neuropathy (assessed by conventional nerve conduction studies) and DR progression; however, the specialized equipment and expertise required limit its practicality. In contrast, DPNCheck^®^ is a simpler alternative that quantifies neuropathy severity through the severity of the estimated modified Baba classification (eMBC).

**Materials and methods:**

Using electronic medical records (EHRs), we identified individuals with diabetes who underwent DPNCheck^®^ and subsequent ophthalmologic assessment for DR. Based on age and sural nerve conduction data, an eMBC was calculated. Meanwhile, DR severity was scored using a modified Davis classification, defining four stages (DR severity scores 0–3).

**Results:**

Of 181 individuals extracted from our hospital’s EHRs, 146 were eligible for analysis. Ordinal logistic regression showed that eMBC was significantly associated with DR stage, independent of diabetes duration and HbA1c. Receiver operating characteristic (ROC) curve analyses yielded eMBC cut-off values of 1.11, 1.51, and 1.51 to predict DR severity scores of ≥1, ≥2, and ≥3, respectively. Sensitivities ranged from 0.67 to 0.78, and specificities from 0.66 to 0.81. An eMBC of 1.51 or above was strongly associated with preproliferative or proliferative DR, indicating a need for urgent ophthalmology referral.

**Conclusions:**

DPNCheck^®^, a simple nerve conduction measurement device, may help predict DR severity and facilitate timely ophthalmologic care.

## Introduction

In 2021, approximately 537 million people worldwide were living with diabetes, and this number is predicted to rise to 783 million by 2045—a substantial global burden in terms of health outcomes, welfare, and healthcare costs (1). Poor glycemic control in diabetes can lead to complications such as diabetic retinopathy (DR), diabetic nephropathy (DN), and diabetic polyneuropathy(DPN), all of which severely affect patients’ quality of life (QOL). Of these complications, DR results from chronic hyperglycemia-induced microvascular and vitreous damage in the retina, causing a range of pathological changes. In the United States, DR is responsible for 5-14% of blindness (2). A meta-analysis involving 22,896 individuals with diabetes across 35 countries reported a global DR prevalence of 35.4% (3). Given the growing number of diabetes cases, there is concern about a parallel increase in DR incidence. In Japan, DR accounts for 10.2% of visual impairment cases (4). Among Japanese adults with type 2 diabetes, the reported annual DR incidence is 3.83%, and the progression rate is 2.11% (5). DR is also recognized as a risk factor for DN, DPN and macrovascular complications (6–8). Therefore, timely detection and accurate assessment of DR severity are vital not only for preserving vision but also for improving long-term survival.

Despite its potential severity, DR often remains asymptomatic until advanced stages. Individuals with diabetes may only notice visual impairment after macular edema, vitreous hemorrhage, or tractional retinal detachment develops, by which time urgent therapeutic interventions are required. Elevated HbA1c levels and a diabetes duration exceeding five years are known to increase the risk of DR (5). In Japan, people with diabetes are advised to undergo at least one fundus examination per year; more frequent exams are recommended if DR is already present, glycemic control is inadequate, or diabetes duration exceeds 10 years (9, 10). Despite these recommendations, only 40-50% of people with diabetes receive regular ophthalmologic care (11). Strengthening systems that encourage ophthalmology visits is thus critical for preventing DR progression.

According to the Japanese Clinical Practice Guideline for Diabetes 2024, individuals with mild DPN should undergo nerve conduction studies (NCS) every 6 months to a year; those with more advanced DPN require more frequent testing to assess progression of DPN more precisely (12). Based on this guideline, we hypothesized that if DR severity could be inferred from routinely monitored DPN severity, it might be possible to prioritize ophthalmology referrals for high-risk individuals. Indeed, studies have reported correlations between DR severity and DPN severity when assessed by the Baba classification (13, 14). The Baba classification is a widely utilized method in Japan for evaluating the severity of diabetic neuropathy, particularly distal symmetric polyneuropathy (12). This system relies on the results of nerve conduction studies (NCS) to provide an objective and highly reproducible assessment of neuropathy progression in individuals with diabetes, categorized into four stages. However, the Baba classification requires NCS, which depends on specialized equipment and trained healthcare professionals with technical expertise (12). As a result, its implementation can be challenging in medical facilities lacking the necessary resources, limiting its widespread adoption. Recently, the simpler and more accessible DPNCheck^®^ device, used in the estimated modified Baba classification (eMBC), has shown good correlation with DPN severity (15). We reasoned that this finding could be leveraged to create a powerful referral tool for ophthalmology.

We further speculated that if DR progression could be anticipated using eMBC values derived from DPNCheck^®^, it might prompt more frequent and timely ophthalmology visits among individuals with diabetes who receive their routine diabetes care from general practitioners rather than diabetes specialists. This could facilitate earlier detection of DR and help prevent severe vision loss. Accordingly, this study aimed to validate a prediction score for DR staging using eMBC values derived from DPNCheck^®^.

## Materials and methods

Study Design and Population. In this single-center retrospective observational study, we included individuals aged 16 years or older with diabetes who underwent DPNCheck^®^ testing while attending our departments at Gifu University Hospital, and whose DR was evaluated by ophthalmologists using the modified Davis classification. The DR evaluation needed to occur within three months before or after DPNCheck^®^ between January 1, 2019, and September 30, 2023.

We excluded those who did not have a DR evaluation within the specified window, those whose nerve conduction velocity (NCV) or amplitude could not be measured by DPNCheck^®^, those who with peripheral neuropathy other than DPN, and those who opted out of study participation. The Gifu University Graduate School of Medicine’s ethics review board approved this study (Approval no. 2021-A057), which was conducted in accordance with the principles of the Declaration of Helsinki, with opt-out informed consent.

Data Collection. We collected baseline characteristics (e.g., age, sex, body mass index [BMI], diabetes duration, smoking status), comorbidities, laboratory data, and medication history from Gifu University Hospital’s electronic medical records (EMRs) for analysis. Macrovascular complications were defined as a history of stroke, coronary artery disease, or peripheral vascular disease. Dyslipidemia and hypertension were defined by the presence of pertinent medication use or relevant physician diagnoses in the EMRs. Laboratory data (e.g., HbA1c, urinary albumin-to-creatinine ratio [ACR], 24-hour urinary albumin, and estimated glomerular filtration rate [eGFR]) obtained within one month before or after DPNCheck^®^ were used. Data on resting CV_R-R_ which was measured within three months before or after DPNCheck^®^ were used. DPNCheck^®^ was used to measure sural NCV and amplitude bilaterally, with the average of these measurements included in the analysis. Instead of the Baba classification, which relies on NCS requiring expensive equipment and specialized technicians, we utilized the eMBC. This alternative demonstrates a strong correlation with the Baba classification and can be easily calculated using sural nerve conduction data obtained via DPNCheck^®^ as follows (15):


eMBC=2.046+0.509×ln(Age [years])−0.033×(Nerve Conduction Velocity [m/s])−0.622×ln(Sensory Nerve Action Potential Amplitude[µV])

DR severity was assessed using the retinopathy severity score (RSS): no DR=0, simple DR=1, preproliferative DR=2, and proliferative DR=3

Statistical Analysis. Baseline characteristics were summarized as median (interquartile range) for continuous variables and frequencies for categorical variables. The Kruskal-Wallis’s test was used to compare continuous variables across RSS categories, and the chi-square test was used for categorical variables. An ordinal logistic regression model was employed to assess the association between eMBC and RSS, adjusting for potential confounders (diabetes duration and HbA1c). Subsequently, binary logistic regression models with the same covariates were run to investigate the relationship between eMBC and each binary outcome (RSS ≥1, RSS ≥2, and RSS ≥3). Receiver operating characteristics (ROC) curve was generated to assess predictive performance and to identify eMBC cut-off values for each DR severity category, using the Youden Index to determine the optimal cut-offs. Sensitivities and specificities were calculated for each. All statistical analyses were conducted using R version 4.4.1 (https://www.r-project.org/), with two-sided p-values <0.05 considered significant.

## Results

**Figure 1:**
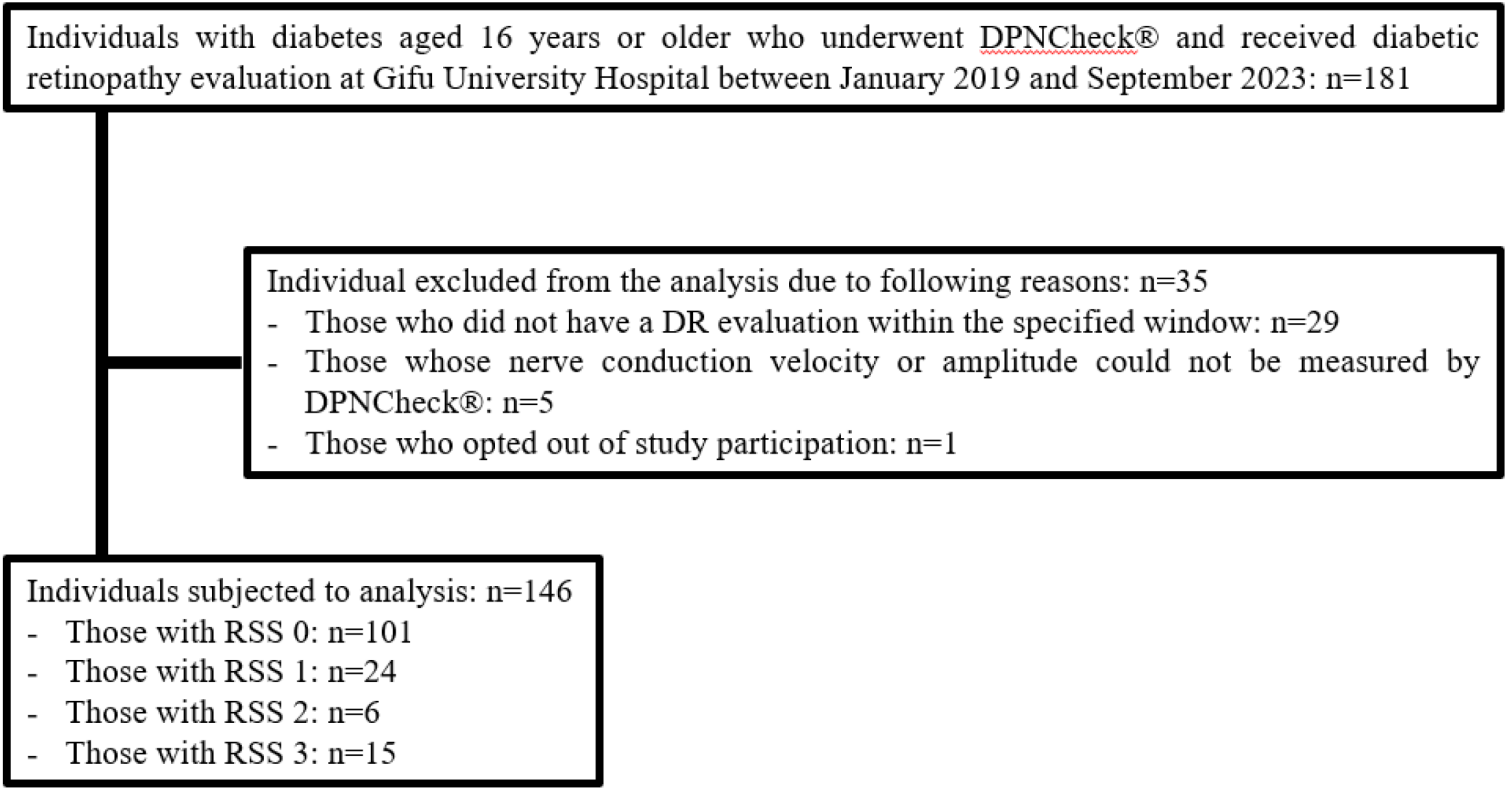
Flowchart of the participants and exclusions in this study. Among 181 individuals aged 16 and older with diabetes who underwent both DPNCheck^®^ and eye examinations at Gifu University Hospital between January 2019 and September 2023, 35 were excluded based on exclusion criteria. The breakdown of excluded individuals is as follows: 29 individuals did not undergo retinopathy evaluation within 3 months before or after the DPNCheck^®^, 5 individuals could not be detected by the DPNCheck^®^, and 1 individual had an unknown duration of diabetes. The final analysis included 146 individuals, categorized as follows: 101 individuals with an RSS of 0 points, 24 with 1 point, 6 with 2 points, and 15 with 3 points.

Among 181 individuals with diabetes who underwent DPNCheck^®^ and received a DR evaluation at Gifu University Hospital, 29 were excluded because the DR evaluation did not occur within the three-month window before or after DPNCheck^®^, 5 were excluded because NCV or amplitude could not be measured, and 1 was excluded because his diabetes diagnosis could not be confirmed from the EMR (**Figure 1**). Ultimately, 146 individuals with complete data were included in the analysis (**Table 1**). Their median age was 69 years; 95 were men, and 51 were women. The median BMI was 24.6 kg/m², and the median diabetes duration was 10 years. Macrovascular complications were present in 25.3% of the individuals analyzed, and approximately half had dyslipidemia or hypertension. The median HbA1c was 8.0%. The median NCV was 50.5 m/s, amplitude was 9.5 µV, and resting CV_R-R_ was 2.3%. The median ACR was 11.3 mg/g, and the median eGFR was 72.2 mL/min/1.73m². Biguanides (43.2%) were the most used glucose-lowering agents, followed by DPP-4 inhibitors (35.6%), insulin (32.2%), and SGLT2 inhibitors (31.5%). Among the 6 patients who received anti-VEGF therapy, the underlying conditions were age-related macular degeneration in 1 patient (RSS 1 point), glaucoma in 1 patient (RSS 0 points), and diabetic macular edema in 4 patients (1 with RSS 2 points and 3 with RSS 3 points). Duration of diabetes, CV_R-R_, ACR, insulin use, GLP-1 receptor agonist use, NCV, amplitude and eMBC differed significantly across RSS groups.

**Table 1:** Clinical characteristics, laboratory results, and neuropathy-related findings of individuals with diabetes included in the current study.

	All	RSS 0	RSS 1	RSS 2	RSS 3	p-value
N	146	101	24	6	15	
Clinical characteristics						
Age (years)	69 (52, 74)	69 (54, 74)	70 (56, 76)	67 (58, 69)	50 (45, 65)	0.138
Male (%)	65.1	60.4	75.0	100	66.7	0.154
Duration of diabetes (years)	10.0 (2.0, 18.5)	8.5 (1.0, 15.0)	10 (7.0, 23.5)	12.5 (9.5, 20)	12.0 (8.5, 21.0)	0.024
BMI (kg/m^2^)	24.6 (22.5, 27.7)	24.6 (21.5, 27.7)	24.3 (22.3, 26.6)	25.9 (24.3, 27.9)	25.7 (23.3, 27.6)	0.504
Macrovascular complications (%)	25.3	20.8	37.5	50.0	26.7	0.178
Hypertension (%)	52.1	49.5	54.2	50.0	66.7	0.660
Hyperlipidemia (%)	50.0	50.5	41.7	66.7	53.3	0.703
Smoking history (%)*	52.6	43.6	62.5	83.3	40.0	0.241
Laboratory findings						
HbA1c (%)*	8.0 (7.2, 9.7)	7.9 (7.0, 9.3)	8.9 (7.8, 11.0)	8.7 (7.1, 9.3)	8.5 (7.7, 9.4)	0.116
ACR (mg/g)*	11.3 (6.4, 28.5)	9.65 (6.0, 21.0)	15.8 (10.2, 45.1)	15.6 (12.3, 405,0)	23.7 (9.5, 116.0)	0.029
eGFR (mL/min/1.73m^2^)	72.2 (57.8, 86.5)	74 (63.9, 88.1)	61.2 (50.9, 80.3)	69.3 (58.8, 81.3)	66.2 (36.9, 88.3)	0.103
Neuropathy-related findings						
Resting CV_R-R_ (%)*	2.30 (1.4, 3.5)	2.45 (1.58, 3.70)	2.32 (1.38, 3.54)	1.90 (1.85, 3.42)	1.67 (1.22, 2.00)	0.047
DPNCheck^®^ NCV (m/s)	50.5 (47.0, 55.5)	52.0 (48.5, 56.0)	49.0 (44.0, 51.8)	48.8 (44.8, 50.1)	45.5 (40.0, 50.0)	<0.001
DPNCheck^®^ amplitude (μV)	9.5 (5.5, 13.9)	11.5 (7.0, 14.5)	6.5 (5.3, 8.5)	5.3 (3.4, 10.1)	3.5 (3.0, 9.8)	<0.001
eMBC	1.10(0.70, 1.51)	0.96 (0.65, 1.35)	1.42 (1.16, 1.68)	1.66 (1.15, 1.68)	1.73 (0.99, 2.05)	<0.001
Anti-diabetes medications						
Insulin use (%)	32.2	27.8	29.1	83.3	46.7	0.022
GLP-1RA use (%)	19.9	19.8	12.5	83.3	6.70	0.039
DPP-4 inhibitors	35.6	34.7	41.7	16.7	40.0	0.80
Sulfonylureas	8.90	10.9	4.20	0	6.70	0.93
Glinides	8.90	8.90	16.7	0	0	0.82
Biguanide	43.2	43.6	37.5	66.7	40.0	0.74
Thiazolidinedione	1.40	2.00	0	0	0	1.00
α-glucosidase inhibitors	12.3	14.9	12.5	0	0	0.77
SGLT2 inhibitors	31.5	28.7	29.1	83.3	33.3	0.17

* As some individuals included in the study lacked non-essential information for inclusion, the number of individuals analyzed for the following items is as follows: Smoking history (All, 133; RSS0, 92; RSS1, 21; RSS2, 6; RSS3, 14), HbA1c (All, 145; RSS0, 101; RSS1, 24; RSS2, 6; RSS3, 14), resting CV_R-R_ (All, 125; RSS0, 85; RSS1, 21; RSS2, 5; RSS3, 14) and ACR (All, 130; RSS0, 92; RSS1, 23; RSS2, 3; RSS3, 12). ACR, albumin-creatinine-ratio; BMI, body mass index; NCV, nerve conduction velocity; CV_R-R_, Coefficient of variation of R-R interval; DPP-4, dipeptidyl peptidase-4; eMBC, estimated modified Baba’s classification; eGFR, estimated glomerular filtration rate; GLP-1RA, glucagon-like peptide-1 receptor agonist; RSS, retinopathy severity score; SGLT2, sodium-glucose cotransporter 2.

Ordinal logistic regression was performed to explore the relationship between eMBC and RSS, adjusting for diabetes duration and HbA1c, as initially hypothesized. Even after accounting for these factors, eMBC remained significantly associated with DR severity (**Table 2**).

**Table 2:** Ordinal logistic regression analysis of the association between retinopathy severity scores and the estimated modified Baba classification.

Variable	Adjusted Odds ratio	95%CI	p-value
eMBC	3.32	1.78-6.20	<0.001
HbA1c (%)	1.15	0.96-1.38	0.119
Duration of diabetes (years)	1.05	1.01-1.08	0.011

eMBC, estimated Baba classification.

**Figure 2:**
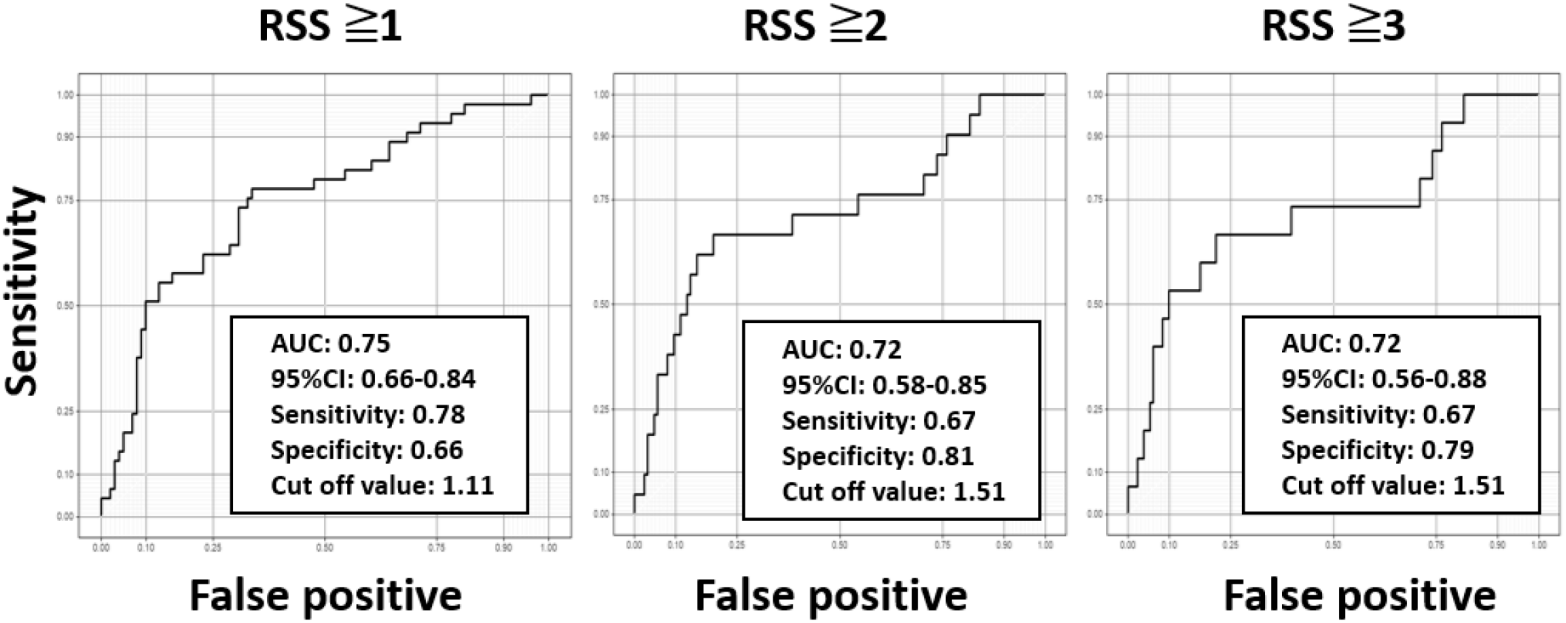
Receiver operating characteristic curves for determining cut-off values for predicting diabetic retinopathy stages using the estimated modified Baba classification with DPNCheck^®^. Using the receiver operating characteristic curves, the optimal estimated modified Baba classification (eMBC) cutoff values for retinopathy severity score (RSS) ≥1, ≥2, and ≥3 were calculated as 1.11, 1.51, and 1.51, respectively. The area under the curve (AUC), 95% confidence interval (CI), sensitivity, and specificity for each cutoff value are indicated in the graphs.

Next, binary logistic regression models (adjusted for diabetes duration and HbA1c) were used to analyze whether eMBC was significantly associated with each binary outcome (RSS ≥1, ≥2, and ≥3) (**Table 3**). We then plotted ROC curves to estimate eMBC cut-off values for each DR severity category (**Figure 2**). For RSS ≥1, ≥2, and ≥3, the optimal eMBC cut-offs were 1.11, 1.51, and 1.51, respectively (with ROC-AUCs of 0.75, 0.72, 0.72). The sensitivity for these respective thresholds was 0.78, 0.67, and 0.67, while specificity was 0.66, 0.81, and 0.79.

**Table 3:** Binary logistic regression analysis of the association between retinopathy severity scores and the estimated modified Baba classification.

	Variable	Odds ratio	95%CI	p-value
RSS ≥1	eMBC	3.47	1.73-6.94	<0.001
	HbA1c (%)	1.21	1.00-1.47	0.052
	Duration of diabetes (years)	1.05	1.01-1.10	0.012
RSS ≥2	eMBC	2.68	1.28-5.58	0.009
	HbA1c (%)	0.98	0.75-1.28	0.883
	Duration of diabetes (years)	1.03	0.99-1.08	0.813
RSS ≥3	eMBC	2.65	1.18-5.97	0.019
	HbA1c (%)	1.01	0.74-1.38	0.938
	Duration of diabetes (years)	1.04	0.99-1.09	0.160

RSS, retinopathy severity score; eMBC, estimated Baba classification.

## Discussion

Our findings indicate that eMBC—calculated from DPNCheck^®^ measurements—can help predict DR severity. This aligns with earlier reports showing a correlation between DR severity and DN severity, as assessed by the Baba classification (13, 14). Although traditional risk factors for DR include longer diabetes duration, higher HbA1c, hypertension, and microalbuminuria (6, 16–19), our study highlighted significant differences in duration of diabetes, eMBC, CV_R-R_, and ACR across DR severity categories, whereas differences in HbA1c and hypertension were not significant. Large-scale clinical trials have found considerable variation in the onset and progression of DR, suggesting that factors beyond established risks (e.g., glycemic variability and genetic predisposition) may influence DR progression (16, 20, 21). Further research is needed to determine how these additional factors affect DR onset and progression. Notably, individuals with high eMBC values showed the progression of DR, regardless of HbA1c levels or the duration of diabetes. This suggests that high eMBC values may be an indicator of the progression of not only DPN due to chronic hyperglycemia, but also DR. Chronic hyperglycemia–induced cellular metabolic abnormalities are currently hypothesized to represent a shared pathophysiological mechanism underlying both peripheral nerve damage and retinal vascular pathology. These abnormalities include activation of the polyol pathway, increased activity of protein kinase C, enhanced flux through the hexosamine pathway, elevated oxidative stress, and upregulation of the advanced glycation end product (AGE) pathway (22). As a consequence, capillary basement membrane thickening and endothelial cell hyperplasia occur in both neural and vascular tissues. These histological changes contribute to the development of diabetic microvascular complications, such as axonal degeneration and demyelination in peripheral nerves, and DR in the retina (23). eMBC has been reported to be a useful indicator of diabetic polyneuropathy (15, 24, 25). In the present study, significant differences in eMBC and ACR were also observed across DR severity classifications, suggesting that eMBC may additionally serve as an indirect marker of microvascular complications such as retinopathy and nephropathy. Although the importance of regular ophthalmologic follow-up and prompt DR treatment is broadly recognized, many people with diabetes do not visit ophthalmologists regularly because of the need for pharmacological mydriasis and the associated time-consuming tests. Delayed DR treatment can lead to severe, sometimes irreversible, visual impairment. In Japan, discontinuation or failure to attend ophthalmic evaluations remains a key obstacle to effective DR management (26). Individuals with diabetes who receive care from non-diabetes specialists are particularly susceptible to missing or skipping ophthalmology visits due to less awareness of DR risks (26). Conversely, those who have lived with diabetes for a longer time may better understand DR risks and thus adhere more consistently to follow-up schedules. This underscores the need for targeted education on DR risks among those with shorter disease duration, including younger people with diabetes.

In this study, eMBC cut-off values for predicting DR severity were identified. An eMBC ≥1.11 signifies a heightened likelihood of simple DR or worse, indicating that such individuals should be strongly encouraged to seek ophthalmologic assessment. More crucially, an eMBC ≥1.51 points to a high risk of severe, vision-threatening DR, highlighting the need for timely treatments such as panretinal photocoagulation and/or intravitreal anti-VEGF therapy. Predicting DR severity based on DPNCheck^®^ readings could serve as a powerful trigger for more urgent ophthalmologic consultations, particularly for individuals who otherwise might not prioritize regular ophthalmologic follow-up.

Innovations in DR detection technology are progressing rapidly; for instance, ultra-widefield scanning laser ophthalmoscopy, which does not require pharmacological mydriasis, can capture detailed images of peripheral retinal regions quickly (27). However, patients must visit an ophthalmology clinic to benefit from this technology. By contrast, DPNCheck^®^ is relatively affordable, easy to administer by non-specialists, and well-suited for primary care settings. These characteristics make it an attractive tool for identifying individuals with diabetes who might be at higher risk of DR and in need of more specialized ophthalmic evaluation. Notably, the Japanese Clinical Practice Guideline for Diabetes 2024 recommends conducting NCS every 6 to 12 months (12); thus, regular use of DPNCheck^®^ at similar intervals is strongly encouraged.

Our study has several limitations. This study has several limitations. First, as the study was conducted at a single center in Japan, the generalizability of the findings to other countries and ethnic groups is limited. Indeed, previous reports have indicated that DPNCheck^®^ measurements may vary by ethnicity (28). Therefore, multicenter and international validation studies are needed to confirm and refine the cutoff values for use in non-Japanese populations. This study examined only the cross-sectional correlation between eMBC and RSS and did not assess the potential of eMBC to predict DR progression or clinical outcomes. We believe that a longitudinal study is warranted to evaluate whether changes in eMBC are associated with the progression of DR. Second, because DPNCheck^®^ can deliver a maximum stimulus of 70 mA, individuals with foot edema or significantly thickened skin due to obesity may have undetectable amplitudes—potentially reflecting more advanced DPN and DR. Third, the cut-off values for RSS ≥2 and RSS ≥3 were both 1.51 in this study, reflecting the limited number of individuals in these more severe stages. Nevertheless, identifying RSS ≥2 remains clinically critical as it is a threshold for earlier ophthalmic intervention. Fourth, standard nerve conduction studies (NCS) were not performed concurrently with DPNCheck^®^ measurements in this study, primarily due to limitations related to insurance coverage. As a result, we were unable to evaluate the correlation between NCS findings and DR severity. Future studies should include direct comparisons between NCS and DPNCheck^®^ to further validate the utility of eMBC in this context. Fifth, in this study, only 6 out of 146 individuals received anti-VEGF therapy, which limited our ability to evaluate its effects on eMBC values. Regarding the impact of anti-VEGF agents on neural tissue, both neuroprotective effects (29) and potential adverse effects (30) have been reported, and a clear consensus has yet to be reached. Further longitudinal studies comparing DPNCheck^®^ results before and after anti-VEGF therapy are warranted to clarify its influence on peripheral nerve function. A key strength of our investigation is that it showcases the potential of a straightforward, cost-effective system using DPNCheck^®^ to approximate DR severity, presenting a practical strategy to address barriers to regular fundus examinations.

In conclusion, DPNCheck^®^, a simple nerve conduction measurement device, may help predict DR severity and facilitate timely ophthalmologic care. Integrating DPNCheck^®^ into routine diabetes care could be instrumental in preventing the progression of DR, ultimately preserving vision and improving long-term outcomes.

## Data Availability

The raw data supporting the conclusions of this article will be made available by the authors, without undue reservation.
