# Supplementary material for: Predicting diabetic retinopathy stages using a simple nerve conduction measuring device, DPNCheck®: a retrospective observational study
Source: Front Clin Diabetes Healthc. 2025 Jul 16;6:1590407. doi: 10.3389/fcdhc.2025.1590407 (PMC12307143; doi:10.3389/fcdhc.2025.1590407)
Supplement: Supplementary file 2 [file Table1.docx]

#----------#

#Library----

#----------#

library(rms)

library(dplyr)

library(knitr)

library(ggplot2)

library(pROC)

library(plotROC)

library(gtsummary)

library(flextable)

library(scales)

library(ggsci)

#--------#

# Data----

#--------#

path <- "【解析依頼】DPNcheckと網膜症20231130_3内科酒井.csv"

data = read.csv(path ,na.strings="",fileEncoding = "SJIS")

data = data %>%

mutate(eMBC_cat = ifelse(eMBC>=1.3065,"DPN進行群","DPN非進行群"),

網膜症スコア0_123 = ifelse(RSS %in% c(1,2,3), 1, 0),

網膜症スコア01_23 = ifelse(RSS %in% c(2,3), 1, 0),

網膜症スコア012_3 = ifelse(RSS %in% c(3), 1, 0),

log_糖尿病罹病期間 = log(罹病期間),

log_ＨｂＡ１ｃ = log(誘発筋電図時ＨｂＡ１ｃ)

)

#-----------#

# Table1.----

#-----------#

summary_table = data %>%

select(性別,年齢,糖尿病診断時年齢,罹病期間,神経伝導速度平均,振幅平均,誘発筋電図時ＨｂＡ１ｃ,

身長,体重,BMI,CVR.R,アルブミン尿,尿蛋白定性,eGFR,

大血管合併症,高血圧症,脂質異常症,喫煙,インスリン,GLP.1A,

SGLT.2i,DPP.4i,BG,ピオグリタゾン,SU,グリニド,α.GI) %>%

tbl_summary(digits = list(everything() ~ c(1),

all_categorical() ~ c(0,1),

c(年齢,糖尿病診断時年齢) ~ c(0)),

type = 尿蛋白定性 ~ "continuous",

missing="no") %>%

add_n() %>%

modify_header(label = "**Variable**")%>%

bold_labels() %>%

as_flex_table()

summary_table

#-----------#

# Table2----

#-----------#

dd = datadist(data)

options(datadist = "dd")

L1 = lrm(RSS ~ eMBC + 誘発筋電図時ＨｂＡ１ｃ + 罹病期間, data = data, x=T, y=T)

p_value = round(anova(L1)[-nrow(anova(L1)),3],3)

p_value = as.character(p_value)

p_value = ifelse(p_value=='0','<0.001',p_value)

sum = summary(L1, eMBC = c(0,1), 誘発筋電図時ＨｂＡ１ｃ = c(0,1), 罹病期間 = c(0,1))

s1 = sum[ 1:(nrow(sum) %/% 2 ) * 2 - 1, c(1)]

name = names(s1)

s2 = round(sum[ 1:(nrow(sum) %/% 2 ) * 2, c(4,6,7)],2)

OR_all = cbind(name,s2,p_value)

colnames(OR_all) = c('Variable','Odds Ratio','95%LCI','95%UCI','p')

rownames(OR_all) = NULL

kable(OR_all)

#------------#

# Table3a.----

#------------#

dd = datadist(data)

options(datadist = "dd")

L1 = lrm(網膜症スコア0_123 ~ eMBC + 誘発筋電図時ＨｂＡ１ｃ + 罹病期間, data = data, x=T, y=T)

p_value = round(anova(L1)[-nrow(anova(L1)),3],3)

p_value = as.character(p_value)

p_value = ifelse(p_value=='0','<0.001',p_value)

sum = summary(L1, eMBC = c(0,1), 誘発筋電図時ＨｂＡ１ｃ = c(0,1), 罹病期間 = c(0,1))

s1 = sum[ 1:(nrow(sum) %/% 2 ) * 2 - 1, c(1)]

name = names(s1)

s2 = round(sum[ 1:(nrow(sum) %/% 2 ) * 2, c(4,6,7)],2)

OR_all = cbind(name,s2,p_value)

colnames(OR_all) = c('Variable','Odds Ratio','95%LCI','95%UCI','p')

rownames(OR_all) = NULL

kable(OR_all)

#------------#

# Table3b.----

#------------#

dd = datadist(data)

options(datadist = "dd")

L1 = lrm(網膜症スコア01_23 ~ eMBC + 誘発筋電図時ＨｂＡ１ｃ + 罹病期間, data = data, x=T, y=T)

p_value = round(anova(L1)[-nrow(anova(L1)),3],3)

p_value = as.character(p_value)

p_value = ifelse(p_value=='0','<0.001',p_value)

sum = summary(L1, eMBC = c(0,1), 誘発筋電図時ＨｂＡ１ｃ = c(0,1), 罹病期間 = c(0,1))

s1 = sum[ 1:(nrow(sum) %/% 2 ) * 2 - 1, c(1)]

name = names(s1)

s2 = round(sum[ 1:(nrow(sum) %/% 2 ) * 2, c(4,6,7)],2)

OR_all = cbind(name,s2,p_value)

colnames(OR_all) = c('Variable','Odds Ratio','95%LCI','95%UCI','p')

rownames(OR_all) = NULL

kable(OR_all)

#------------#

# Table3c.----

#------------#

dd = datadist(data)

options(datadist = "dd")

L1 = lrm(網膜症スコア012_3 ~ eMBC + 誘発筋電図時ＨｂＡ１ｃ + 罹病期間, data = data, x=T, y=T)

p_value = round(anova(L1)[-nrow(anova(L1)),3],3)

p_value = as.character(p_value)

p_value = ifelse(p_value=='0','<0.001',p_value)

sum = summary(L1, eMBC = c(0,1), 誘発筋電図時ＨｂＡ１ｃ = c(0,1), 罹病期間 = c(0,1))

s1 = sum[ 1:(nrow(sum) %/% 2 ) * 2 - 1, c(1)]

name = names(s1)

s2 = round(sum[ 1:(nrow(sum) %/% 2 ) * 2, c(4,6,7)],2)

OR_all = cbind(name,s2,p_value)

colnames(OR_all) = c('Variable','Odds Ratio','95%LCI','95%UCI','p')

rownames(OR_all) = NULL

kable(OR_all)

#-------------#

# ROC curve----

#-------------#

f.roc <- function(var, outcome, lab) {

eval(parse(text=paste("groc <- ggplot(data, aes(m = ",var,", d = ",outcome,")) + geom_roc(pointsize = 0,labels = F)

p_all_s <- groc+style_roc(xlab='偽陽性',ylab='感度') + theme(panel.grid.major.x = element_line(colour ='grey60'),panel.grid.major.y = element_line(colour ='grey60'),legend.title=element_blank(),legend.position='top')

p_all_s <- p_all_s+ ggtitle('",lab,"')

ROC <- roc(",outcome," ~ ",var,", data = data)

AUC <- round(ROC$auc,2)

ci.auc <- paste(round(ci.auc(ROC)[1],2),'-',round(ci.auc(ROC)[3],2),sep='')

threshold <- coords(ROC, 'best', ret=c('sensitivity', 'specificity','threshold'), best.weights=c(1, 0.5))

threshold <- round(as.numeric(threshold[c(1,2,3)]),2)

Res_ROC <- c('",lab,"',AUC,ci.auc,threshold[1],threshold[2],threshold[3])

", sep="")))

return(list(Res_ROC,p_all_s))

}

res1 = f.roc("eMBC","網膜症スコア0_123","網膜症スコア0/123")[[1]]

res2 = f.roc("eMBC","網膜症スコア01_23","網膜症スコア01/23")[[1]]

res3 = f.roc("eMBC","網膜症スコア012_3","網膜症スコア012/3")[[1]]

Res_roc_all = rbind(res1,res2,res3)

colnames(Res_roc_all) = c('Factor','AUC','95%CI','Sensitivity','Specificity','Threshold')

rownames(Res_roc_all) = NULL

kable(Res_roc_all)

ROC1 <- f.roc("eMBC","網膜症スコア0_123","網膜症スコア0/123")[[2]]

ROC1

ROC2 <- f.roc("eMBC","網膜症スコア01_23","網膜症スコア01/23")[[2]]

ROC2

ROC3 <- f.roc("eMBC","網膜症スコア012_3","網膜症スコア012/3")[[2]]

ROC3
